# Regulatory fine-tuning of *mcr-1* increases bacterial fitness and stabilises antibiotic resistance in agricultural settings

**DOI:** 10.1038/s41396-023-01509-7

**Published:** 2023-09-18

**Authors:** Lois Ogunlana, Divjot Kaur, Liam P. Shaw, Pramod Jangir, Timothy Walsh, Stephan Uphoff, R. C. MacLean

**Affiliations:** 1 Department of Biology, University of Oxford, 11a Mansfield Road, Oxford, OX1 3SZ UK; 2 Department of Biosciences, Durham University, Stockton Road, Durham, DH1 3LE UK; 3 Ineos Oxford Institute for Antimicrobial Research, University of Oxford, South Parks Road, Oxford, OX1 3RE UK; 4 Department of Biochemistry, University of Oxford, South Parks Road, Oxford, OX1 3QU UK

**Keywords:** Population genetics, Antimicrobials

## Abstract

Antibiotic resistance tends to carry fitness costs, making it difficult to understand how resistance can be maintained in the absence of continual antibiotic exposure. Here we investigate this problem in the context of *mcr-1*, a globally disseminated gene that confers resistance to colistin, an agricultural antibiotic that is used as a last resort for the treatment of multi-drug resistant infections. Here we show that regulatory evolution has fine-tuned the expression of *mcr-1*, allowing *E. coli* to reduce the fitness cost of *mcr-1* while simultaneously increasing colistin resistance. Conjugative plasmids have transferred low-cost/high-resistance *mcr-1* alleles across an incredible diversity of *E. coli* strains, further stabilising *mcr-1* at the species level. Regulatory mutations were associated with increased *mcr-1* stability in pig farms following a ban on the use of colistin as a growth promoter that decreased colistin consumption by 90%. Our study shows how regulatory evolution and plasmid transfer can combine to stabilise resistance and limit the impact of reducing antibiotic consumption.

## Introduction

Antibiotic resistance in pathogenic bacteria (AMR) has emerged as a fundamental threat to human health, prosperity, and food security [1–3]. The acquisition of antibiotic resistance by mutation or horizontal gene transfer is usually associated with fitness costs, such as reduced competitive ability and virulence. Given these costs, reducing antibiotic consumption should generate selection against resistance, driving the loss of AMR in pathogen populations [4–9]. Although the logic of this strategy is simple, interventions aimed at reducing antibiotic consumption have often resulted in marginal reductions in the prevalence of resistance, and a key challenge in the field is to understand the ecological and evolutionary processes that allow resistance to be maintained in the absence of continual antibiotic exposure [9–11].

Experimental evolution studies have found that resistance is usually maintained in bacterial populations that are not exposed to antibiotics due to selection for compensatory mutations that offset the cost of resistance. The idea that resistance is stabilised by compensatory adaptation is deeply ingrained in evolutionary models of antibiotic resistance [9, 12–16]. However, direct examples of resistance being stabilised by compensatory adaptation in clinical pathogen populations are lacking [11], with the notable exception of studies on *Mycobacterium tuberculosis* [17, 18]. Moreover, alternative mechanisms can stabilise resistance without compensatory adaptation. For example, resistance genes can persist as a result of selection for linked genes, such as biocide resistance genes, a phenomenon called co-selection in the AMR literature [10]. Many resistance genes are carried on mobile genetic elements, particularly conjugative plasmids, that provide resistance genes with the opportunity to transfer horizontally. High rates of conjugative transfer could allow resistance to persist in pathogen populations, even if the plasmids themselves impose a fitness cost [19–21].

Here we test the hypothesis that compensatory evolution stabilises resistance to colistin, an agricultural antibiotic that is increasingly being used as a ‘last line of defence’ for the treatment of infections caused by multi drug resistant Gram-negative pathogens. The widespread of use of colistin as an animal growth promoter drove the sudden spread of *Escherichia coli* carrying **m**obile **c**olistin **r**esistance (i.e., MCR) genes across one-health settings, including farms, humans and the environment [22]. Many *mcr* homologues have now been identified, but *mcr-1* remains the most prevalent and best-characterised colistin resistance gene [23–25]. The *mcr-1* gene initially spread as part of a composite IS*Apl*1 transposon that transferred between plasmids, which themselves transferred between strains of pathogenic and commensal *E.coli* and other enteric bacteria [26]. The expression of *mcr-1* results in fundamental changes to the bacterial outer membrane, leading to extensive pleiotropic effects [27, 28] that are associated with large fitness costs. For example, expression of *mcr-1* can reduce *E.coli* growth rates by as much as 30% [27].

The Chinese government responded to the spread of *mcr-1* by banning the use of colistin as a growth promoter in animal feed in April 2017, resulting in a 90% reduction in colistin consumption in China [24, 29, 30]. Large-scale surveillance studies across one-health sectors found that the prevalence of *mcr-1* declined following the colistin ban [24, 29], providing strong evidence to support the idea that the spread of *mcr-1* was driven by using colistin in as an animal growth promoter [26]. However, the rate at which the prevalence of *mcr-1* declined following the ban was much slower than would be expected given the reported costs of colistin resistance [27]. For example, the frequency of *mcr-1* carriage in pigs, which were a key reservoir of colistin resistance, declined from 34% pre-ban (2015–16) to 5.1% post-ban (2017–2018) [29]. If we conservatively assume that *E. coli* has a generation time of ~12 h [31, 32], this decline over a two-year period spanning >1000 generations would suggest a fitness cost of <1%. Here we test the hypothesis that compensatory adaptation stabilised colistin resistance in *E.coli* by combining functional assays to measure to fitness effects of *mcr-1* polymorphisms with analysis of large scale genomic and epidemiological datasets.

## Results

### Regulatory polymorphisms alleviate the cost of *mcr-1*

**Fig. 1:**
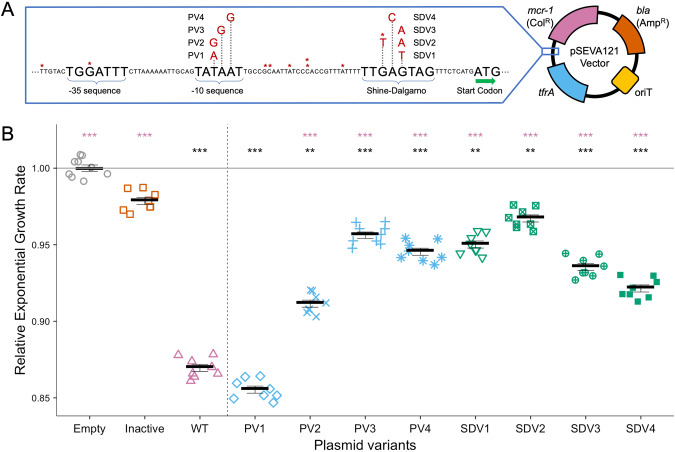
Construction and fitness assessment of *mcr-1* regulatory variants. **A** Schematic shows the region immediately upstream of the *mcr-1* start codon. The wild-type upstream sequence is shown in black. Regulatory regions are labelled, with polymorphisms shown in red. Polymorphisms found in each tested regulatory variant are named. Asterisks denote sites where additional variant SNPs (not tested in this study) were found with each asterisk representing one variant SNP. Regulatory variants and a WT regulatory sequence were cloned into the pSEVA121 vector. **B** Relative exponential growth rates in colistin-free media are shown for the WT regulatory sequence (WT, pink) and promoter (blue) and Shine-Dalgarno (green) variants. Empty vector (grey) and inactivated *mcr-1* (orange) controls are included. Empty vector growth rate is set to 1 (solid grey line). The experiment was replicated over eight different days. Each plotted point shows the average growth rate of five replicate cultures in a single run of the experiment. Significance of comparisons to empty vector (black) and WT (pink) controls are indicated (*p* values: ***<0.001. Dunnett’s *t* test adjusted for multiple comparisons, Error bars = standard error, *n* = 5).

A comprehensive genomic analysis of MCR positive *E. coli* published in 2018 identified a polymorphism hotspot upstream of the *mcr-1* gene [26]. Examination of this region revealed the presence of multiple polymorphisms in regions predicted to encode RNA polymerase binding (i.e. −10 and −35 boxes) and Shine-Dalgarno (SD) sites (Fig. 1A, Table S1). The presence of parallel nucleotide substitutions in these regulatory elements suggests that positive selection has occurred to alter *mcr-*1 expression [33]. To directly test this hypothesis, we cloned *mcr-1* into pSEVA121, a mini-RK2 derived expression vector, under the control of either the wild-type (WT) regulatory sequence (i.e. the consensus sequence), or one of eight known ‘regulatory variant’ sequences (Fig. 1A). These variants were each associated with a single polymorphism within the −10 promoter or SD region (apart from SDV2 which had two polymorphisms within the SD region). The pSEVA121 vector used in our experiments has a similar copy number (~4–6 per cell) to natural plasmids that carry *mcr-1* (typically 2–5 per cell [34]). A further advantage of using pSEVA121 plasmids is that they have high stability, even in the absence of selection for plasmid encoded resistance markers (Fig. S1).

To test the impact of regulatory polymorphisms on fitness, we first measured the growth rates of regulatory variants and wild-type strains in colistin-free culture medium, which is a common method to assess the fitness costs of resistance [35]. As a control, we also measured the fitness effect of an inactivated variant which carried a wild-type regulatory sequence and a mutated *mcr-1* active site (T285A) [36, 37]. Seven of the eight regulatory variants were associated with increased growth rates relative to strains possessing the wild-type *mcr-1* regulatory sequence, providing clear evidence that regulatory polymorphisms reduce the cost of colistin resistance (Fig. 1B).

### Regulatory polymorphisms reduce *mcr-1* expression and activity

In silico analysis revealed that all constructed regulatory polymorphisms are predicted to reduce *mcr-1* transcription and/or translation (Table S2), suggesting a simple link between reduced MCR-1 abundance and increased fitness. The -10 sequence of *mcr-1* is a perfect match to the canonical *E. coli σ*^*70*^-10 RNA polymerase binding sequence TATAAT [38–41]. Three of the four variants reduce the AT-richness of this site, a feature known to facilitate DNA melting and aid transcription initiation [42, 43], suggesting that these mutations should decrease *mcr-1* expression. SD sequences are less definitively characterised, but well-established *E. coli* ribosome binding sites are often purine-rich [44]. Tested variants (apart from SDV3) contain purine to pyrimidine substitutions, suggesting that these mutations reduce translation efficiency.

**Fig. 2:**
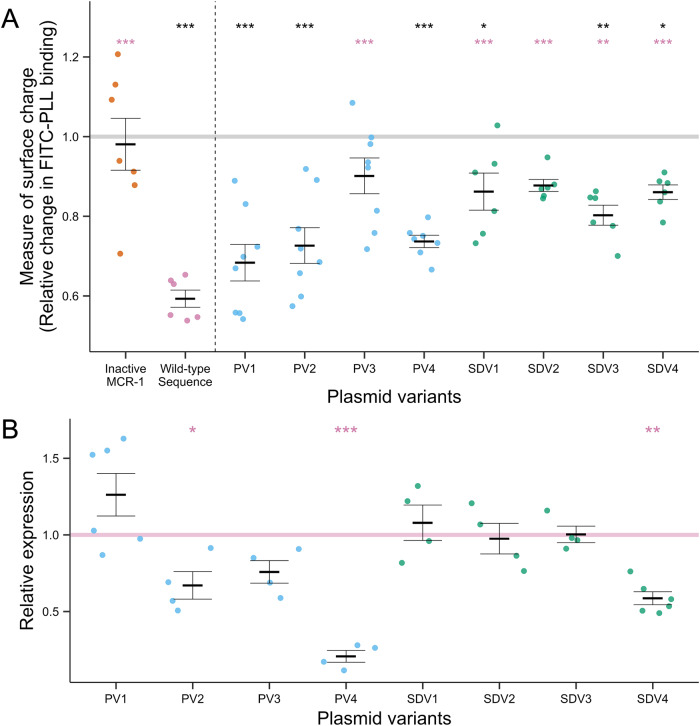
Activity and expression assessments of regulatory variants. **A** MCR-1 activity was assessed by measuring relative cell surface charge using a FITC-PLL binding assay. Surface charges of regulatory *mcr-1* variants (PV = blue, SDV= green), inactivated *mcr-1* (orange) WT sequence (pink) were measured relative to an empty control (grey line, set to 1). Significance in comparison to the empty vector (black) and consensus expression (pink) are indicated above the respective plasmid variants (*p* values: ***<0.001, **<0.01, *<0.05, Dunnett’s *t* test adjusted for multiple comparisons, *n* = 6–10, error bars = SE) **B** Relative *mcr-1* transcript levels of variants (PV = blue, SDV= green) are shown standardised to the WT regulatory sequence (pink line, set to 1). Solid variant lines show mean relative expression, points individual values. Significance in comparison to WT are indicated above the respective variants (*p* values: ***<0.001, **<0.01, *<0.05, ns=not significant. Dunnett’s t-test adjusted for multiple comparisons, *n* = 4–6, error bars = SE).

We took two experimental approaches to understand the link between *mcr-1* expression and fitness. The first approach focused on MCR-1 protein activity. Mutating the catalytic site of MCR-1 largely eliminated the cost of *mcr-1* carriage in the absence of colistin, suggesting that the cost of *mcr-1* mainly comes from the activity of the MCR-1 protein (Fig. 1B). Phosphoethanolamine transferase activity of MCR-1 results in a reduction of membrane surface charge due to the neutralisation of negatively charged lipid A phosphate groups on lipopolysaccharide (LPS) molecules [25, 45]. This reduction of cell surface charge is thought to prevent the binding of positively charged colistin to bacterial membranes [46]. To investigate the link between MCR-1 activity and fitness, we measured the impact of regulatory mutations on cell surface charge in the absence of colistin. Expressing *mcr-1* with the WT regulatory sequence reduced cell surface charge, whereas this effect was partially alleviated in regulatory variants, suggesting that regulatory variants reduce the expression of the MCR-1 protein (Fig. 2A). As a second approach, we measured the impact of regulatory variants on levels of *mcr-1* transcription (Fig. 2B). Regulatory variants generally reduced levels of *mcr-1* transcript abundance which is indicative of reduced *mcr-1* transcription and/or increased mRNA degradation. This reduction in expression was particularly evident for regulatory variants with mutations in the *mcr-1* promoter region (i.e., the -10 sequence), consistent with the idea that the cause of decreased expression is reduced transcription initiation.

### Regulatory fine-tuning increases colistin resistance and fitness

**Fig. 3:**
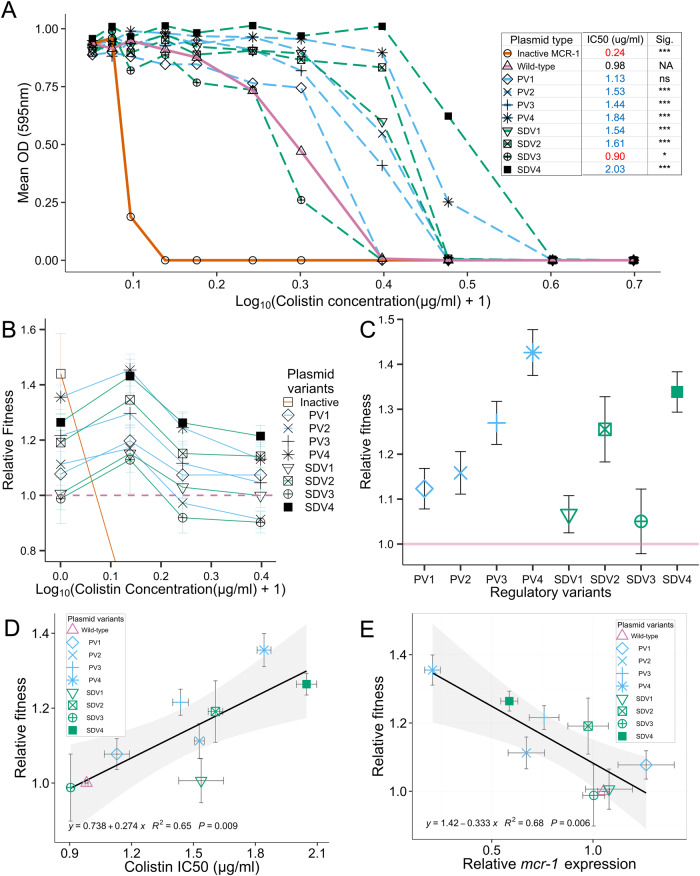
Resistance and fitness assessments of regulatory variants. **A** Mean OD in a range of colistin concentrations (0–2 µg/ml, Log_10_ Scale) is shown for regulatory variants (dashed lines, PV = blue, SDV = green) and controls (solid lines, pink = WT, grey = Empty control), *n* = 15. Estimated colistin IC50s of control and variants are shown, as determined by fitting OD data to a dose-response model. IC50 values that are lower and higher than the WT highlighted in red and blue respectively, with statistical significance determined by pairwise comparisons of variants to the WT regulatory sequence (*p* values adjusted using the Bonferroni correction for multiple comparisons ****<0.001, *<0.05). **B** Relative fitness of regulatory variants and inactivated control compared to the WT control (set at 1, dotted pink line). Fitness values below the lower limit (0.8) are not shown. Error bars = standard error, *n* = 5. **C** Estimated single fitness values for each regulatory variant are plotted (error bars=SE). **D**, **E** Scatterplots showing the relationship between (**D**) Colistin IC50 (*x*-axis) or (**E**) mean expression (*x*-axis) against mean relative fitness in the absence of colistin (y-axis). Points assigned to variants/controls are indicated in the legend (error bars = SE). Linear regression model (black line) was fitted to the data (confidence intervals in grey shading). Regression line equation, model r-squared and *p* value indicated in black text.

Compensatory mutations are generally thought to overcome the cost of antibiotic resistance without compromising resistance [10]. However, given that regulatory variants were associated with decreased *mcr-1* expression and/or MCR-1 activity, we hypothesised that these variants would be associated with a trade-off in terms of decreased fitness in the presence of colistin [47, 48]. As an initial test of this hypothesis, we measured the impact of regulatory mutations on colistin resistance using MIC assays. The colistin resistance of regulatory variants was equal to or greater than that of the WT control strain (Fig. 3A). As a second approach to measure colistin resistance levels, we calculated the IC50 for colistin, which provides a quantitative estimate of the colistin concentration needed to reduce bacterial growth by 50%. Six of the regulatory variants were associated with an increased IC50, and a single variant caused a slight reduction in IC50.

To further examine fitness trade-offs associated with regulatory mutations, we directly measured the impact of regulatory mutations on competitive fitness across a gradient of colistin concentrations by co-culturing *E. coli* possessing regulatory variants and WT control strains (Fig. 3B; Table S5 for statistical models). The strain carrying catalytically inactivated MCR-1 under the control of a wild-type regulatory sequence had high fitness in the absence of colistin and low fitness in the presence of colistin (Fig. 3B). Regulatory variants, on the other hand, were associated with increased fitness in both the presence and absence of colistin, although there was significant variation in fitness between regulatory variants (main effect variant: *p* value = 1.84e-14). Colistin concentration had an impact on the fitness of regulatory variants (main effect [colistin]: *p* value = 1.08e-10), with a maximal fitness advantage under intermediate doses of colistin. However, the impact of colistin concentration did not differ between variants (variant*[colistin] interaction: *p* value = 0.983). Given this, we used our statistical model to estimate a single fitness value for each regulatory variant, and all regulatory variants had higher fitness than the wild-type strain (Fig. 3C).

To better understand the surprising finding that *mcr-1* regulatory mutations increase fitness and colistin resistance, we used a linear regression to test for a correlation between resistance and fitness. In this analysis, we used only fitness data that was collected in colistin-free media to avoid spurious correlations between fitness and colistin resistance. Fitness was strongly correlated with increased resistance, highlighting the ability of regulatory mutations to fine-tune *mcr-1* expression without any associated trade-offs (Fig. 3D; *F*_(1,7)_ = 12.96, *p* value = 0.009, *r*^2^ = 0.65)

Given that regulatory variants had altered *mcr-1* activity and expression, we used multiple regression to independently estimate the contribution of decreased activity and expression to increased fitness. Fitness was strongly correlated with *mcr-1* expression, highlighting the value of optimising *mcr-1* expression (Fig. 3E; *F*_(1,7)_ = 14.76, *p* value = 0.006, *r*^2^ = 0.68). The high fitness of the catalytically inactive MCR-1 mutant compared to the WT reference strain implies that MCR-1 activity confers fitness costs. However, measurements of the MCR-1 activity of the regulatory variant strains (as measured by cell surface charge) were not correlated with fitness, implying that variation in MCR-1 activity was not a consistent source of variation in fitness between the regulatory mutants (Fig. S2).

### Chromosomal *mcr-1* is associated with high fitness and resistance

**Fig. 4:**
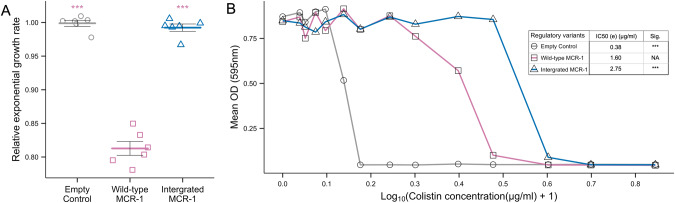
Assessments of *mcr-1* chromosomal integration. **A** Relative exponential growth rates in colistin-free media are shown for the empty vector (grey) *mcr-1* plasmid (pink) and chromosomally integrated *mcr-1* (dark blue). Empty vector growth rate is set at 1. **B** Mean OD in a range of colistin concentrations (0–6 µg/ml, Log_10_ Scale) is indicated for empty vector (grey) plasmid *mcr-1* (pink) and chromosomally integrated *mcr-1* (dark blue). (*n* = 8). Predicted IC50s of empty vector (grey), plasmid (pink) and chromosome integrated (dark blue) wild-type regulatory sequences. Significance shows results of pairwise comparisons of each variant to plasmid localised wild-type regulatory sequence *(p* values adjusted using the Bonferroni correction for multiple comparisons, ***<0.001).

As a final test of the link between *mcr-1* expression and resistance, we manipulated *mcr-1* expression by inserting the *mcr-1* gene under the control of a wild-type regulatory sequence into the *E. coli* chromosome. This manipulation decreased *mcr-1* expression by moving the gene from a multi-copy pSEVA plasmid replicon (4–5 copies/cell) to a single copy chromosomal replicon. Consistent with our earlier results, *E. coli* with a chromosomally integrated *mcr-1* had increased growth rate (Fig. 4A) and colistin MIC (Fig. 4B) compared to the positive control strains in which *mcr-1* was expressed from a low copy number plasmid.

### Evolutionary origins of regulatory variants

*mcr-1* is almost always carried on conjugative plasmids, with the main replicons being IncX4, IncI2 and IncHI2 [23, 24]. To better understand the evolutionary trajectory of *mcr-1* regulation, we re-analysed a published genomic dataset from large-scale surveillance of colistin-resistant *E. coli* from human, environmental, and agricultural sources in China between 2016-2018 (we refer to this as the ‘Shen dataset’) [24]. Our analysis focused on the presence of regulatory variants on plasmids.

The evolutionary model for *mcr-1* spread is well-established. It is believed that *mcr-1* was initially mobilised from *Moraxella* species as a part of a composite IS*Apl1* transposon into *E. coli*, but the IS*Apl1* elements flanking *mcr-1* degenerated following transposition to different plasmid backgrounds. This degeneration resulted in the loss of active transposition and a ‘fossilisation’ of the *mcr-1* cassette in these backgrounds [26, 49]. The presence of repetitive sequences in the IS*Apl1* copies flanking *mcr-1* also leads to fragmented *mcr-1* plasmid assemblies when isolates are sequenced using short-read technologies. Anecdotally, the presence of *mcr-1* on short contigs (<3 kb) due to fragmented assembly is thus suggestive of the intact IS*Apl1* composite transposon.

**Fig. 5:**
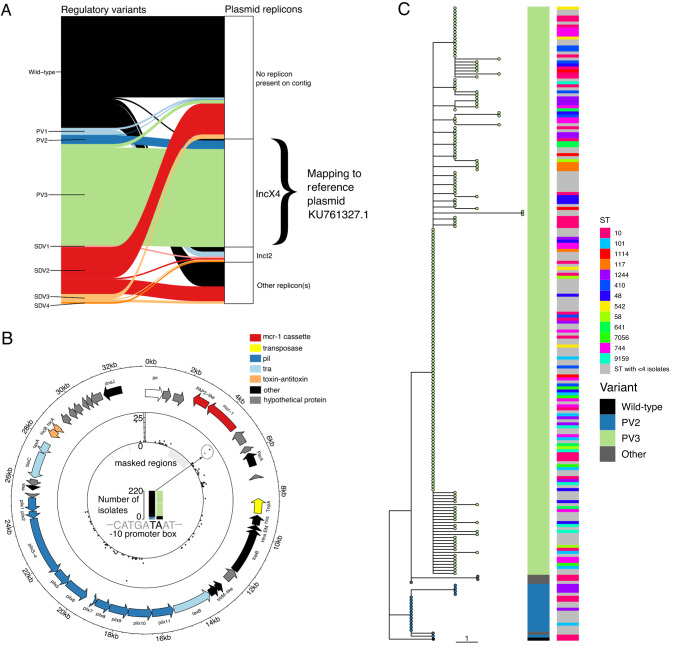
Associations between regulatory variants and plasmid replicons. **A** Sankey diagram of regulatory variants and plasmid replicons present on *mcr-1*-containing contigs (*n* = 569 isolates). Not shown are isolates with 75 bp upstream sequences without an exact match to the consensus or the named regulatory variants (*n* = 110). **B** The 32.6 kb IncX4 plasmid (NCBI KU761327.1) used for reference-based mapping of short-reads from *n* = 220 isolates with *mcr-1* and IncX4 on a contig in their de novo assembly. Inner track shows the number of isolates with a minority variant at that site. The highlighted zoom in the centre of the plot shows the number of isolates with each base at the sites corresponding to the PV2- and PV3-associated SNPs. PV3 is the dominant regulatory variant seen in IncX4. Outer track shows genes after annotation with Prokka and grouping into gene groups. **C** A phylogeny of IncX4 plasmids from reference-based mapping (*n* = 211 after removing poorly mapped plasmids) shows clades of regulatory variants PV2 and PV3 (tip colours, also first column). The phylogeny is rooted to a plasmid with the wild-type regulatory sequence. Scale bar shows 1 SNP. Closely-related IncX4 plasmids with PV3 (top clade) are more prevalent and seen across a huge ST diversity (second column, STs with 4 or more isolates coloured by ST).

In line with this, we found that in the Shen dataset, the wild-type regulatory sequence was most associated with contigs that carried no plasmid replicon, suggesting an association between the WT regulatory sequence and the presence of IS*Apl1* (Fig. 5A). Furthermore, these contigs were shorter on average than contigs associated with regulatory variants (median 27.0 kb vs. 32.6 kb for non-wild-type sequences), providing further evidence to support this idea. The wild-type regulatory sequence was strongly associated with extremely short contigs (39.4% of wild-type regulatory sequences were on contigs <3 kb vs. 12.0% for regulatory variants, *n* = 569 isolates, Chi-squared test *X*^2^ = 55.9, *p* < 0.001), consistent with regulatory variants being associated with ‘fossilised’ *mcr-1* sequences that lack copies of IS*Apl1*.

We observed strong associations between regulatory variants and plasmid replicons (Fig. 5A). Most notably, there was a strong association between IncX4 and regulatory variant PV3: 194/220 (88.2%) of IncX4-containing contigs also had PV3, and 194/201 (96.5%) PV3-containing contigs also had IncX4. No IncX4-containing *mcr-1*-containing contigs had an intact copy of IS*Apl1*.

To obtain greater phylogenetic resolution, we re-mapped reads from isolates with IncX4-containing *mcr-1*-containing contigs against a reference *mcr-1*-positive IncX4 plasmid first observed in 2014 (KU761327.1, Fig. 5B; see Methods). Although the genetic diversity of these IncX4 plasmids was low, a phylogeny showed that PV2 and PV3 plasmids are in two different clades, suggesting that regulatory fine-tuning in IncX4 occurred at least twice and separately (Fig. 5C). One IncX4-containing plasmid carried the wild-type regulatory sequence, consistent with a scenario where an ancestral IncX4 plasmid acquired a mobile form of *mcr-1* (i.e. with IS*Apl1* present) followed by the loss of IS*Apl1* and subsequent regulatory evolution: in one instance to PV2, in another to PV3. We found that the regulatory variants were already well-established in isolates from 2016, consistent with these changes arising prior to 2016 (Fig. S3). An alternative scenario would be three independent acquisitions of the *mcr-1* region with different regulatory variants (wild-type, PV2, PV3).

The presence of compensatory mutations on a conjugative plasmid generates a worst-case scenario for resistance management, as it creates the potential for low-cost AMR plasmids to transfer between host strains [50]. Indeed, plasmids with the PV3 regulatory variant were seen across a broad diversity of host strains in the Shen dataset (*n* = 71 STs, Fig. 5C). This highlights how a conjugative plasmid with a fine-tuned resistance gene can rapidly disseminate low-cost resistance across diverse strains. While it is also possible that PV3 was repeatedy and independently acquired by near-identical plasmids in different STs, this seems unlikely: there is a strong association between plasmid replicons and regulatory mutations and even within IncX4 the regulatory variants form clear clades (Fig. 5C). The most common host strain for the IncX4 plasmid was ST10 (*n* = 26 isolates), a known and prevalent livestock-associated extra-intestinal pathogenic *E. coli* lineage [51] that has been proposed to play a key role in the dissemination of *mcr-1* plasmids [52].

### Regulatory mutations stabilise colistin resistance

**Fig. 6:**
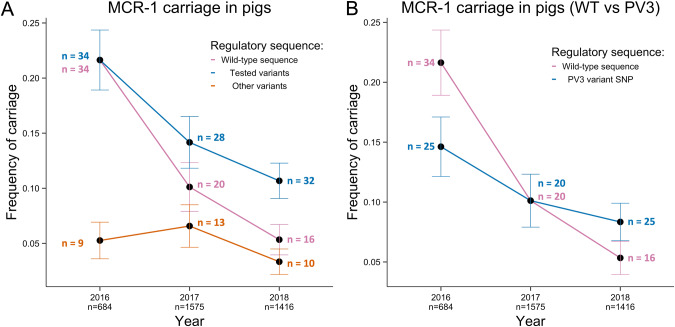
Stability of regulatory variants on pig farms. **A** Frequency of carriage was determined for wild-type (pink) and variant (blue) and untested variant (orange) sequences across the years for pig samples (n shows the total number of pigs sampled each year (below x axis) and the number of isolates with each mcr-1 variant (within figure)). Each pig was treated as an independent unit. Error bars show the propagated standard error. **B** Frequency of carriage of wild-type (pink) sequences compared to those carrying PV3 (blue) variant SNPs. Error bars show the propagated standard error.

To test the impact of regulatory mutations on *mcr-1* stability, we calculated changes in the prevalence of regulatory polymorphisms before (2016) and after (2017, 2018) colistin was banned as growth promoter using data from pig farms, which were intensively sampled due to the importance of farms as a source of colistin resistance (66 farms, 684–1575 pigs per year). One advantage of focusing on pig farms is that combining our genomic analysis with data on the prevalence of *mcr-1* carriage in pigs allowed us to estimate changes in the carriage rate of *mcr-1* variants over time. The proportion of tested regulatory variants increased between 2016 and 2018 relative to WT sequences (Fig. 6A, *p* value = 0.037; one-sided two-sample test for equality of proportions without continuity correction, Fig. S5), reflecting an increased stability of regulatory variants in pigs compared to *mcr-1* with a wild-type regulatory sequence. Interestingly, the increase in the prevalence of tested regulatory variants in comparison to the total population of *mcr-1*-positive isolates was marginal (*p* value = 0.11: one-sided two-sample test for equality of proportions without continuity correction), suggesting that the other variants may also carry compensatory mutations.

A closer look at the genomic data from pig farms revealed that the variant population was dominated by PV3, which was associated with clear increases in fitness across colistin concentrations (Fig. 3). As we would expect, the frequency of PV3 increased compared to WT following the colistin ban (Fig. 6B, *p* value = 0.033; one-sided two-sample test for equality of proportions without continuity correction). Crucially, the prevalence of the PV3 variant in pigs remained more stable following the colistin ban, suggesting that the benefits of this mutation are sufficient to offset the costs associated with *mcr-1* on pig farms.

## Discussion

The rapid spread of *mcr-1*-mediated colistin resistance in *E. coli* represents an important threat to human health given that colistin provides a ‘last line of defence’ for the treatment of infections caused by multi-drug resistant *E. coli*. Here we show that the *mcr-1* regulatory region has evolved to reduce the cost of *mcr-1* while simultaneously increasing colistin resistance, providing a poignant demonstration of the ability of mutation and natural selection to fine-tune gene expression (Figs. 1–3). Conjugative plasmids carrying these *mcr-1* regulatory variants have transferred between a diversity of host strains, disseminating this ‘low-cost/high-resistance’ phenotype across *E. coli* (Fig. 5). Crucially, regulatory variants were associated with increased *mcr-1* stability in pigs following a ban on the use of colistin as a growth promoter that reduced colistin use in agriculture by 90% (Fig. 6). These findings provide a clear and unambiguous example of how the adaptive evolution of resistance genes together with plasmid transfer can stabilise antibiotic resistance and limit the impact of reducing antibiotic consumption.

One common model for the horizontal transfer of resistance genes is that composite transposons mobilise resistance genes from the chromosomes of commensal bacteria to plasmids that are then transferred to pathogenic bacteria [26, 48, 53]. For example, *mcr-1* is thought to have been mobilised from the chromosome of pig-associated bacteria by a IS*Apl*1 composite transposon that subsequently transferred *mcr-1* to plasmids associated with *Enterobacteriaceae* [26]. An important consequence of this pathway of gene mobilisation is that resistance genes are overexpressed in their new bacterial hosts as plasmid typically have higher copy number than the chromosome. For example, the dominant plasmids replicons that carry *mcr-1* in *E.coli* (e.g. IncX4, IncI2, IncHI2) typically have copy numbers of 2–8 [27, 34].

The over-expression of resistance genes from multi-copy plasmids is likely to generate fitness costs. In the case of *mcr-1*, this cost reflects the deleterious consequences of modifications to the bacterial cell surface mediated by MCR-1, and not the cost of MCR-1 protein synthesis per se (Fig. 2A). Increasing the copy number of resistance genes often increases antibiotic resistance, such that high antibiotic doses generate selection for chromosome-to-plasmid transposition of resistance genes [47, 48]. *mcr-1* provides an interesting counterexample to this pattern, as the high levels of *mcr-1* expression generated by multi-copy plasmids actually lead to decreased colistin resistance (Fig. 3A). Our study was not able to unravel the mechanistic basis of this result, but the tight correlation between fitness and colistin resistance (Fig. 3D) suggests that high levels of MCR-1 result in membrane modifications, such as increased cell permeability [27], that are deleterious in both the absence of colistin and during high colistin stress. According to this view, colistin resistance is maximised by intermediate levels of *mcr-1* expression that reduce cell surface charge enough to prevent colistin from efficiently binding to bacterial membrane whilst at the same time minimising the pleiotropic effects of MCR-1 activity on membrane structure and fitness.

The classical model for compensatory adaptation in antibiotic resistant bacteria is that compensatory mutations overcome the cost of resistance without altering antibiotic susceptibility [10]. Mutations in the regulatory region of *mcr-1* provide a simple mechanism to fine-tune the expression of this gene, increasing both fitness and colistin resistance (Fig. 3B–E). The high copy number of *mcr-1* plasmids is likely to have facilitated regulatory evolution by increasing the mutation rate of this gene [47]. An important consequence of this evolutionary trajectory is that conjugative plasmids allow low-cost/high-resistance alleles of *mcr-1* to transfer between strains of *E.coli* with different niches (Fig. 5C). We speculate that gene mobility plays a key role in stabilising *mcr-1* in the face of perturbations, such as altered antibiotic use, that favour a sub-set of *E.coli* strains [54]. For example, chromosomal integration of the *mcr-1* transposon has occurred [55] and our results suggest that this is likely to have led to increased resistance at low cost. However, the low prevalence of chromosomally integrated *mcr-1* [24, 26] suggests that this simple pathway for ameliorating the cost of resistance is an evolutionary dead end. An important challenge for future work will be to directly test the importance of horizontal gene transfer in stabilising antibiotic resistance in pathogen populations [20, 56]. This may be especially important in a one-health context where HGT can allow resistance genes to spread across strains associated with human, agricultural and environmental niches [57–59].

Experimental evolution studies have found that a wide range of genetic mechanisms can compensate for the cost of acquiring costly plasmids [60]. Mutations in chromosomal regulatory proteins that modulate the expression of plasmid or chromosomal genes are probably the most common mechanism of compensatory evolution [61–65]. Plasmids carrying AMR genes also impose differential costs across bacterial hosts [21, 35, 66] suggesting that chromosomal variation is key to shaping the cost of plasmid carriage. In this case, colistin resistance is shaped by the interplay between *mcr-1* and chromosomal genes involved in LPS biosynthesis [67], and it is possible that mutations in chromosomal genes also contribute to stabilising *mcr-1*. Plasmid mutations outside of the *mcr-1* regulatory region may have also contributed to offsetting the cost of *mcr-1*. For example, the IS*Apl1* transposon that mobilised MCR-1 contains a putative promoter [68], suggesting that the initial degeneration of the transposon may have been driven by selection to minimise the cost of *mcr-1*.

One of the simplest interventions to combat AMR is to reduce antibiotic consumption, and the ban on the use of colistin as a growth promoter represents one of the largest and best studied attempts to combat resistance using this approach. In many respects, the evolution of *mcr-1* towards increased resistance and fitness and on-going transmission of *mcr-1* across *E. coli* strains represents a worst-case scenario for resistance management. However, it is important to emphasise that the prevalence of *mcr-1* continued to decline across one-health sectors following the ban on use of colistin as a growth promoter [24, 29]. Additionally, regulatory variant alleles which increased bacterial fitness and *mcr-1* stability still declined in overall frequency as the result of the ban. This study showcases the importance of continued resistance gene surveillance and sequence monitoring following antibiotic use bans. The key implication of these findings for resistance management is that accurately forecasting the impact of reducing antibiotic consumption on AMR may need to consider the evolution and transmission of resistance genes. Our findings suggest that antibiotic use bans can successfully reduce the prevalence of partially compensated alleles, albeit less effectively compared to the original costly resistance gene sequences. However, in extreme cases where resistance has negligible costs and/or a very high rate of transfer, limiting antibiotic consumption may not be a viable strategy to reduce resistance.

## Methods

### Strains, plasmids, and growth conditions

All experiments were carried out in *E. coli* MG1655 using Luria-Bertani or MHB medium (Sigma-Aldrich). All control and constructed plasmids are listed in Supplementary Table S3. Media was supplemented with colistin (Cayman Chemical) or ampicillin (Sigma-Aldrich) as appropriate to enable selected growth of plasmid carriers and for MIC assays.

### Oligonucleotides

A full list of DNA oligonucleotides used in this work is provided in Table S4. All oligonucleotides were ordered from ThermoScientific.

### *mcr-1* regulatory variant construction

A synthetic pSEVA vector containing *mcr-1* and its natural promoter as a cargo was constructed. *mcr-1* and its surrounding regions (75 bp upstream, 40 bp downstream) were PCR-amplified from the PN16 (IncI2) plasmid using Q5 High-Fidelity DNA Polymerase (New England BioLabs). The amplified fragment was cloned into pSEVA121 using the NEBuilder HiFi DNA Assembly kit (New England BioLabs) as per manufacturer’s instructions. Plasmids containing one of the eight regulatory variants and inactivated (T285A) *mcr-1* were generated using mutagenic primers and the Q5 Site-Directed Mutagenesis Kit New England BioLabs) as per manufacturer’s instructions. Similar mutagenic primers were used to introduce unique qPCR sequence tags for each variant and control plasmid. Sequence verified plasmids were transformed into MG1655 *E. coli* strains.

### Construction of chromosomally Integrated *mcr-1*

MG1655 with chromosomally integrated *mcr-1* was constructed by lambda red recombineering. The consensus *mcr-1* gene and regulatory sequences were cloned into the MG1655 chromosome in replacement of the non-essential *lacZ* gene. Isolates were confirmed for correct assemblies using blue-white screening and sequence verification.

### Resistance determination via minimum inhibitory concentration (MIC) assays

Colistin resistance of constructed strains was determined using standard broth microdilution methods and OD measurements. Bacteria were grown in MHB media supplemented with the appropriate antibiotics (50 µg/ml ampicillin for MG1655 containing pSEVA plasmids and 1 µg/ml colistin for MG1655 containing chromosomally integrated *mcr-1*). Bacteria were diluted to 5 × 10^5^ CFU/ml in a range of colistin concentrations (Cayman Chemical, 0–8 µg/ml) in alternating two-fold dilutions. Eight independent replicates were performed for all strains and concentrations. Bacteria were grown in 96-well culture plates (Nunc MicroWell 96-Well Microplates) overnight (37 °C, 250 rpm). Readings were done using the BioTek Synergy 2 plate reader. OD measurements (595 nm) were taken following incubation with subtraction of media background measurements. OD values below 0.1 were determined to have no bacterial growth with the lowest concentration where this was reached determined as the MIC. IC50 values were estimated by fitting dose response curve (DRC) models to MIC data using the R-studio software and the drc package for dose-response curve analysis (v2.5-12, https://rdocumentation.org/packages/drc/versions/2.5-12. All models obtained non-significant scores using lack-of-fit tests.

### Growth rate measurements

Overnight cultures of constructed and/or control MG1655 were diluted to 5 × 10^5^ CFU/ml in MH media. Bacteria were incubated overnight at 37 °C with OD measurements (595 nm) taken every 10 min. Regression lines were fitted onto the exponential phases of growth curves. Exponential growth rate (mOD/min) was determined as the maximum slope obtained over ten OD measurement points. Experiments were repeated five times per variant on eight different days. Growth rates varied systematically between assays carried out on different days, as judged by an ANOVA including a main effect of assay day, and we corrected for this by using residual growth rates after correcting for the effect of assay day.

### Gene expression measurements using RT-qPCR

*mcr-1* expression levels were quantified in MG1655 containing control or regulatory variant plasmids. Total RNA was extracted from bacteria following incubation in MH supplemented with Ampicillin (50 µg/ml). Bacteria were sub-cultured, grown to an OD of 0.5 (595 nm) and washed in phosphate-buffered saline (PBS). Bacterial digestion was performed using RNAprotect Bacteria Reagent (Qiagen) as per manufacturer’s instructions. Digested bacteria were subjected to RNA extraction using the RNeasy Mini Kit (Qiagen) and QiaCube liquid handling platform (Qiagen) as per manufacturer’s instructions. Extracted RNA was treated using the TURBO DNA-free kit (ThermoFisher) and quantified using the Quantifluor RNA system (Promega).

Extracted RNA was diluted to 5 µg/ml and subjected to one step RT-qPCR on the StepOnePlus Real-Time PCR System (Applied Biosystems) using the Luna Universal One-Step RT-qPCR Kit (New England Biolabs). *mcr-1* and t*rfA* (reference) expression were quantified using appropriate primers (Supplementary Table 4). *trfA* was selected as a reference due to its pSEVA121 localisation allowing normalisation of plasmid copy number across all control and regulatory variant plasmids tested. Plasmid standard curves were used to estimate primer efficiencies of *mcr-1* (103%) and *trfA* (103.3%) primers. Three technical replicates were performed for each biological replicate variant tested. CT values obtained were all within the quantifiable range of template. Fold change in expression between each variant and the consensus *mcr-1* regulatory was calculated using the following equation that considers primer efficiencies.

$${Fold}\,{Change}=\frac{{E}_{{ref}}^{({{Ct}}_{{variant}}-{{Ct}}_{{consensus}})}}{{E}_{{target}}^{({{Ct}}_{{variant}}-{{Ct}}_{{consensus}})}}$$

Where *E*_ref_ is the *trfA* primer efficiency, *E*_target_ is the *mcr-1* primer efficiency and Ct refers to the cycle thresholds obtained for variant and consensus plasmids using the appropriate primers.

### Bacterial surface charge measurements

Fluorescent isothiocyanate-labelled poly-L-lysine (FITC-PLL, Sigma) binding assays were used to determine *mcr-1* activity. Positively charged FITC-PLL can bind Gram-negative outer membrane in a charge dependant manner due to negative charges on lipid A. *mcr-1* activity reduces these negative charges allowing estimations of *mcr-1* activity by measuring cell fluorescence. Overnight cultures of constructed and/or control MG1655 were washed and 1X PBS buffer to a final OD595 of 0.1. FITC-PLL solution was added to re-suspended cells (5 µg/ml) and samples were incubated at room temperature for 12 min. Following centrifugation (6000 *g*, 5 min), fluorescence measurements (Ex-500 nm/Em-530 nm) of supernatants were compared with PBS controls to determine the proportion of bacteria-bound dye.

### qPCR fitness competitions

Primers were designed to match unique sequence tags on control and variant plasmids to determine plasmid concentrations via specific amplification (Supplementary Table 4). Specificity and optimisation tests were performed for all primers using PCR and qPCR. For competitions, overnight MG1655 cultures carrying control or regulatory variant pSEVA plasmids were OD normalised and diluted to pooled mixes (5 × 10^5^ CFU/ml total). Mixes were used to inoculate MH media of four conditions (0, 0.375, 0.75 and 1.5 µg/ml colistin) and competitions were carried out over a 20 h incubation at 37 °C. Four independent replicates were performed per competition. Resultant competition media and inoculation mixes were boiled (100 °C, 20 min) and qPCR was carried out using the StepOnePlus Real-Time PCR System (Applied Biosystems). Variant specific primers were used to obtain plasmid specific CTs. Standard curves were generated for each primer pair to allow determination of variant plasmid concentrations in each competition.

Relative fitness was calculated for each condition using the following equation.

$${Relative}\,{Fitness}={\log }_{2}\left(\frac{{Vf}}{{Vi}}\right)/{\log }_{2}\left(\frac{{Cf}}{{Ci}}\right)$$

Where Vf = variant plasmid concentration following competition, Vi = variant plasmid concentration of initial pools, Cf = consensus *mcr-1* plasmid concentration following competition and Ci = consensus *mcr-1* plasmid concentration of initial pools. An ANOVA was fitted to the fitness data including effects of variant, colistin concentration (categorical variable or continuous variable) and a variant*colistin concentration interaction term. Note that this model did not include data from the variant with a catalytically inactivated MCR-1.

Linear regression models were fitted to variant relative fitness vs colistin concentration data. Y-intercepts of the resulting models were used as single estimated fitness values for the different regulatory variants.

### Genomic datasets

#### Dataset

We downloaded *n* = 688 paired-end short-read sequencing datasets for *mcr-1*-positive *E. coli* isolates from Shen et al. 2020 (NCBI BioProject PRJNA593695). These isolates were collected between 2016-2018 from pigs, humans (both healthy volunteers and hospital inpatients), food and the environment in Guangzhou, China. We trimmed adaptors with Trimmomatic v0.39 then de novo assembled isolates with Spades v3.15.3 (-k 21,33,55,77, otherwise default parameters). Processed data and scripts are available on github (https://github.com/liampshaw/mcr1-regulatory-variants; commit 997a7c8) and archived on figshare (10.6084/m9.figshare.20943256).

#### Analysing regulatory variants

We wrote custom python scripts to extract *mcr-1*-containing regions from de novo assemblies, detect plasmid replicons using ABRicate, and classify variants in the promoter region. These scripts are packaged as the conda package ‘mcroni’ v1.0.4 (see: https://github.com/liampshaw/mcroni). To assign regulatory variants, we required an exact match to the 75 bp upstream sequences used in experiments (consensus and the eight named variants: PV1-4 and SDV1-4.). Sequences that did not exactly match a named sequence were categorised as “other” (*n* = 110 from de novo assemblies).

#### IncX4 plasmid analysis

After de novo assembly and analysis with mcroni, we selected *n* = 220 isolates which had an IncX4 replicon on their *mcr-1*-containing contig. The median length of these contigs was 32,643 bp (IQR: 32,641-32,850 bp, range: 9331-40,975 bp). We then used a reference-mapping approach to construct a phylogeny of closely-related IncX4 plasmids. To select a suitable reference plasmid, we searched PLSDB v2021_06_23_v2 using a de novo assembled 32.6 kb plasmid from isolate SRR15732044. We chose an *mcr-1*-containing IncX4 plasmid (mash similarity >0.998) isolated from a *K. pneumoniae* isolate from peritoneal fluid from a hospital inpatient in China in September 2015 (KU761327.1). The same study also found an identical plasmid in an *E. coli* isolate from a separate patient in August 2014. We mapped reads to this reference with snippy v4.6.0 (--mincov 10, --minfrac 0.9, otherwise default parameters) which uses bwa and Freebayes. We then used snippy-core to identify core SNPs, masking a region containing the remnants of the *mcr-1*-containing cassette from the start of *mcr-1* (bases 2337-4762 inclusive positions with respect to KU761327.1; note that this does not mask the upstream *mcr-1* regulatory region so regulatory variation is included in the alignment) and two 152 bp regions with a repeated CDS which meant that reads did not map uniquely (29959-30111, 32039-32191). This repeated CDS had a BLAST hit to a YajB protein (AIF97194.1; e-score = 4e-14, 88% query cover, 77.3% identity).

Nine isolates had unaligned positions (range: 9-10,512 unaligned sites after masking) which could indicate either incomplete sequencing or different IncX4 backgrounds. We removed these isolates leaving *n* = 211 isolates. Consensus plasmid sequences from mapped reads had a median of 1 SNP against the reference IncX4 plasmid (range: 0–4). Although we would not expect a temporal signal, this corresponds to an estimated mutation accumulation rate of 0.3 substitutions per plasmid per year, or ~1e-5 substitutions per site per year. We produced a phylogeny with FastTree v2.1 (flags: -nt -gtr) and rooted it to the single plasmid among the *n* = 211 that had the wild-type regulatory sequence (SRR15731853). For plotting, we used ggplot2 [69] v3.3.6, cowplot [70] v1.1.1, ggsankey v0.0.99999 (Fig. 4A, github.com/davidsjoberg/ggsankey), circlize [38] v0.4.15 (Fig. 4B), and ggtree [71] v3.2.1 and ggtreeExtra [72] v1.4.2 (Fig. 5C, Fig. S3). Data and scripts for this analysis are available on figshare (10.6084/m9.figshare.20943256).

### Frequency of carriage calculations

Frequency of carriage of variant and consensus sequences were obtained by multiplying frequency of *mcr-1* prevalence with frequency of variant prevalence. *mcr-1* prevalence data in pigs and hospitalised patients were obtained from Supplementary Tables 1 and 2, respectively. Previous published sequence data from the Shen dataset [24] were analysed to obtain the frequency of variant prevalence. 95% confidence intervals were obtained for both frequencies. The propagated uncertainty for multiplication was calculated using the following equation:

$$\frac{\Delta {{{{{\rm{z}}}}}}}{z}=\sqrt{{\left(\frac{\Delta x}{{{{{{\rm{x}}}}}}}\right)}^{2}+{\left(\frac{\Delta y}{{{{{{\rm{y}}}}}}}\right)}^{2}}$$

Standard error values were obtained from 95% confidence intervals using the following equation:

$${SE}=({Upper}\,{limit}-{lower}\,{limit})/3.92$$

Data was visualised using ggplot. We tested for a difference in the frequency of variants by comparing the proportion of isolates with a designated variant to the proportion of isolates with a wild-type regulatory sequence using the normal approximation to the binomial. We compared regulatory variant frequency for pig populations in 2016 (pre-ban) to the frequency in 2018 (post-ban) as a proportion of all tested regulatory sequences and as a proportion of all sequenced *mcr-1* positive pig isolates. We also compared the PV3 variant frequency for pig populations in 2016 (pre-ban) to the frequency in 2018 (post-ban) as a proportion of PV3 and WT sequences and as a proportion of all sequenced *mcr-1* positive pig isolates. Two-sample tests for equality of proportions were conducted one-sided and without Yate’s correction for continuity.

### Supplementary information

Supplemental figures and tables

## Data Availability

All data reported in this paper is publicly available via the Oxford Research Archive at 10.5287/ora-7r46wn298.
