# Supplementary material for: Regulatory fine-tuning of mcr-1 increases bacterial fitness and stabilises antibiotic resistance in agricultural settings
Source: ISME J. 2023 Sep 18;17(11):2058–69. doi: 10.1038/s41396-023-01509-7 (PMC10579358; doi:10.1038/s41396-023-01509-7)
Supplement: Supplementary file 1 — Supplemental figures and tables [file 41396_2023_1509_MOESM1_ESM.pdf]

Supplementary Material

Tables

Variant Name	Location	Variant Prevalence in Dataset						Sequence
		All Datasets	Wang	Shen (Total)	Shen (2016)	Shen (2017)	Shen (2018)	
Consensus	NA	521	225	268	150	90	29	TTGTACTGGATTTCTTAAAAAATTGCAGTATAATTGCCGCAATTATCCCACCGTTTATTTTTTGAGTAGTTTCTC
PV1	-10 promoter box	78	63	14	4	7	3	TTGTACTGGATTTCTTAAAAAATTGCAGTA**A**AATTGCCGCAATTATCCCACCGTTTATTTTTTGAGTAGTTTCTC
PV2	-10 promoter box	32	14	18	7	8	3	TTGTACTGGATTTCTTAAAAAATTGCAGTA**G**AATTGCCGCAATTATCCCACCGTTTATTTTTTGAGTAGTTTCTC
PV3	-10 promoter box	231	31	200	57	81	29	TTGTACTGGATTTCTTAAAAAATTGCAGTAT**G**ATTGCCGCAATTATCCCACCGTTTATTTTTTGAGTAGTTTCTC
PV4	-10 promoter box	2	2	0	0	0	0	TTGTACTGGATTTCTTAAAAAATTGCAGTATA**G**TTGCCGCAATTATCCCACCGTTTATTTTTTGAGTAGTTTCTC
SDV1	Shine-Dalgarno	22	19	3	0	2	1	TTGTACTGGATTTCTTAAAAAATTGCAGTATAATTGCCGCAATTATCCCACCGTTTATTTTTTGA**T**TAGTTTCTC
SDV2	Shine-Dalgarno	142	41	93	45	35	13	TTGTACTGGATTTCTTAAAAAATTGCAGTATAATTGCCGCAATTATCCCACCGTTTATTTTTT**T**A**A**TAGTTTCTC
SDV3	Shine-Dalgarno	38	22	16	4	10	2	TTGTACTGGATTTCTTAAAAAATTGCAGTATAATTGCCGCAATTATCCCACCGTTTATTTTTTGA**A**TAGTTTCTC
SDV4	Shine-Dalgarno	8	5	3	1	1	1	TTGTACTGGATTTCTTAAAAAATTGCAGTATAATTGCCGCAATTATCCCACCGTTTATTTTTTG**C**GTAGTTTCTC
SNP1	From Mid to SD	58	27	31	15	14	2	TTGTACTGGATTTCTTAAAAAATTGCAGTATAATTGCC**A**CAATTATCCCACCGTTTATTTTTTGA**T**TAGTTTCTC
SNP2	Mid: Between -10 and SD	14	6	8	2	4	2	TTGTACTGGATTTCTTAAAAAATTGCAGTATAATTGCCGCAATTAT**A**CCACCGTTTATTTTTTGAGTAGTTTCTC
SNP3	Mid: Between -10 and SD	2	2	0	0	0	0	TTGTACTGGATTTCTTAAAAAATTGCAGTATAATTGCCG**T**AATTATCCCACCGTTTATTTTTTGAGTAGTTTCTC
SNP4	Mid: Between -10 and SD	4	3	1	0	1	0	TTGTACTGGATTTCTTAAAAAATTGCAGTATAATTGCCGCAATTATCCC**G**CCGTTTATTTTTTGAGTAGTTTCTC
SNP5	Shine-Dalgarno	1	0	1	0	1	0	TTGTACTGGATTTCTTAAAAAATTGCAGTATAATTGCCGCAATTATCCCACCGTTTATTTTTT**A**AGTAGTTTCTC
SNP6	Mid: Between -10 and SD	5	0	5	3	2	0	TTGTACTGGATTTCTTAAAAAATTGCAGTATAATTGCCGCAATTATC**A**CACCGTTTATTTTTTGAGTAGTTTCTC
SNP7	-35 promoter box	13	6	6	0	4	2	TTGTACTG**A**ATTTCTTAAAAAATTGCAGTATAATTGCCGCAATTATCCCACCGTTTATTTTTTGAGTAGTTTCTC
SNP8	-35 promoter box	4	2	2	0	2	0	CTGTACTGGATTTCTTAAAAAATTGCAGTATAATTGCCGCAATTATCCCACCGTTTATTTTTTGAGTAGTTTCTC

Supplementary table 1: Counts of consensus and variant sequences in Shen and Wang datasets. Variant sequences are listed with variants highlighted (blue) in bold underline.

Regulatory Variant	***Promoter Assessments***				***Shine-Dalgarno Assessments***	
	BacPP Score (100)	BacPP Score (75)	BacPP Score (25)	DeNovoDNA Transcription Initiation Rate (au) (100)	DeNovoDNA Translation Initiation Rate (au) (35)	DeNovoDNA Translation Initiation Rate (au) (45 + full *mcr-1*)
Consensus	**72.375**	**86**	**40**	**7419.839125**	**832.0925053**	**708.14**
PV1	69.5625	81	45	5311.77532	-	-
PV2	67.5625	77	36	4313.308475	-	-
PV3	70.4375	77	31	4204.091093	-	-
PV4	69.0625	77	31	4788.265912	-	-
SDV1	-	-	-	-	526.1665373	526.17
SDV2	-	-	-	-	526.1665373	526.17
SDV3	-	-	-	-	526.1665373	526.17
SDV4	-	-	-	-	253.7995844	145.25

Supplementary table 2: Scores obtained for promoter and Shine-Dalgarno using In-Silico analysis. Numbers in brackets indicate query length (in base pairs).

Plasmid	Relevant features	Source/Reference
pSEVA121	AmpR, OriT+	SEVA material repository
pn16	*mcr-1*+, IncI2 plasmid	[27]
pSEVA121:*mcr-1*	consensus *mcr-1* sequence, consensus regulatory sequence	this study
pSEVA121:inactive *mcr-1*	(T285A) *mcr-1* in pSEVA121	this study
pSEVA121:PV1	*mcr-1*.1 + promoter variant 1 + unique tag	this study
pSEVA121:PV2	*mcr-1*.1 + promoter variant 2 + unique tag	this study
pSEVA121:PV3	*mcr-1*.1 + promoter variant 3 + unique tag	this study
pSEVA121:PV4	*mcr-1*.1 + promoter variant 4 + unique tag	this study
pSEVA121:SDV1	*mcr-1*.1 + shine-delgarno variant 1 + unique tag	this study
pSEVA121:SDV2	*mcr-1*.1 + shine-delgarno variant 2 + unique tag	this study
pSEVA121:SDV3	*mcr-1*.1 + shine-delgarno variant 3 + unique tag	this study
pSEVA121:SDV4	*mcr-1*.1 + shine-delgarno variant 4 + unique tag	this study

Supplementary table 3: List of plasmids used/created in this study

Primer	Length (bp)	Sequence (5’-> 3’)	Primer Use
p16_F	38	GATAACAATTTCACACAGGAGGATTGCGCAATGATTGC	*mcr-1* amplification
p16_R	41	AGGGTTTTCCCAGTCACGACCATAATACGAATGGAGTGTGC	*mcr-1* amplification
pSEVA_F	21	GTCGTGACTGGGAAAACCCTG	pSEVA121 amplification
pSEVA_R	24	TCCTGTGTGAAATTGTTATCCGCT	pSEVA121 amplification
MisoInF	38	CGTGCGGCACATCGGCGGCGTATTCTGTGCCGTGTATG	*mcr-1* inactivation
MisoInR	38	CATACACGGCACAGAATACGCCGCCGATGTGCCGCACG	*mcr-1* inactivation
Gibson_F	39	GCTTTCTACGTGGCTGCCATTTTTGGGGTGAGGCCGTTC	Regulatory variant SNP substitution
MisoPV1F	51	GTACTGGATTTCTTAAAAAATTGCAGTAAAATTGCCGCAATTATC CCACCG	Regulatory variant SNP substitution
MisoPV1R	51	CGGTGGGATAATTGCGGCAATTTTACTGCAATTTTTTAAGAAATC CAGTAC	Regulatory variant SNP substitution
MisoPV2F	51	GTACTGGATTTCTTAAAAAATTGCAGTAGAATTGCCGCAATTATC CCACCG	Regulatory variant SNP substitution
MisoPV2R	51	CGGTGGGATAATTGCGGCAATTCTACTGCAATTTTTTAAGAAATC CAGTAC	Regulatory variant SNP substitution
MisoPV3F	51	GTACTGGATTTCTTAAAAAATTGCAGTATGATTGCCGCAATTATC CCACCG	Regulatory variant SNP substitution
MisoPV3R	51	CGGTGGGATAATTGCGGCAATCATACTGCAATTTTTTAAGAAATC CAGTAC	Regulatory variant SNP substitution
MisoPV4F	51	GTACTGGATTTCTTAAAAAATTGCAGTATAGTTGCCGCAATTATC CCACCG	Regulatory variant SNP substitution
MisoPV4R	51	CGGTGGGATAATTGCGGCAACTATACTGCAATTTTTTAAGAAATC CAGTAC	Regulatory variant SNP substitution
MisoSDV1 F	50	GCCGCAATTATCCCACCGTTTATTTTTTGATTAGTTTCTCATGATG CAGC	Regulatory variant SNP substitution
MisoSDV1 R	50	GCTGCATCATGAGAAACTAATCAAAAAATAAACGGTGGGATAAT TGCGGC	Regulatory variant SNP substitution
MisoSDV2 F	50	GCCGCAATTATCCCACCGTTTATTTTTTTAATAGTTTCTCATGATG CAGC	Regulatory variant SNP substitution
MisoSDV2 R	50	GCTGCATCATGAGAAACTATTAAAAAAATAAACGGTGGGATAAT TGCGGC	Regulatory variant SNP substitution
MisoSDV3 F	50	GCCGCAATTATCCCACCGTTTATTTTTTGAATAGTTTCTCATGATG CAGC	Regulatory variant SNP substitution
MisoSDV3 R	50	GCTGCATCATGAGAAACTATTCAAAAAATAAACGGTGGGATAAT TGCGGC	Regulatory variant SNP substitution
MisoSDV4 F	50	GCCGCAATTATCCCACCGTTTATTTTTTGCGTAGTTTCTCATGATG CAGC	Regulatory variant SNP substitution
MisoSDV4 R	50	GCTGCATCATGAGAAACTACGCAAAAAATAAACGGTGGGATAAT TGCGGC	Regulatory variant SNP substitution
TIwt_F	37	TAGAATGGTCGACGTCGTCGTGACTGGGAAAACCCTG	Unique sequence tag insertion
TIwt_R	37	CACTTCGCGATTGAGTCATAATACGAATGGAGTGTGC	Unique sequence tag insertion
TIin_F	37	GCCTGCGATATACCGTGTCGTGACTGGGAAAACCCTG	Unique sequence tag insertion
TIin_R	37	TTATCTTACCGGACTGCATAATACGAATGGAGTGTGC	Unique sequence tag insertion
TIpv1_F	37	CGCATGGACACTATTAGTCGTGACTGGGAAAACCCTG	Unique sequence tag insertion
TIpv1_ R	37	AGCGCTCCAGGTCAATCATAATACGAATGGAGTGTGC	Unique sequence tag insertion
TIpv2_F	37	GTCTAATCAGCGACAGGTCGTGACTGGGAAAACCCTG	Unique sequence tag insertion
TIpv2_ R	37	TGACTGCGTACTCTGACATAATACGAATGGAGTGTGC	Unique sequence tag insertion
TIpv3_F	37	AGGAATCATTCGGTCCGTCGTGACTGGGAAAACCCTG	Unique sequence tag insertion
TIpv3_ R	37	CATCTCGAATGCTGGTCATAATACGAATGGAGTGTGC	Unique sequence tag insertion
TIpv4_F	37	CTGGCAACGCAGTCAGGTCGTGACTGGGAAAACCCTG	Unique sequence tag insertion
TIpv4_ R	37	ATCGAGGTAAGGATAACATAATACGAATGGAGTGTGC	Unique sequence tag insertion

TIsdv1_F	37	CCTGAGCAGCCTGATCGTCGTGACTGGGAAAACCCTG	Unique sequence tag insertion
TIsdv1_ R	37	ATGCACTACTCCTAATCATAATACGAATGGAGTGTGC	Unique sequence tag insertion
TIsdv2_F	37	TGATCGACCGAGAGCTGTCGTGACTGGGAAAACCCTG	Unique sequence tag insertion
TIsdv2_ R	37	GCCGTAATTTAGAGTCCATAATACGAATGGAGTGTGC	Unique sequence tag insertion
TIsdv3_F	37	ATATGTGGTGCAGCCAGTCGTGACTGGGAAAACCCTG	Unique sequence tag insertion
TIsdv3_ R	37	TATCAGGCGACATGAGCATAATACGAATGGAGTGTGC	Unique sequence tag insertion
TIsdv4_F	37	TGAGAGTCGCAACTGTGTCGTGACTGGGAAAACCCTG	Unique sequence tag insertion
TIsdv4_ R	37	GAAGGTTGTTCCAACCCATAATACGAATGGAGTGTGC	Unique sequence tag insertion
tWT	32	ACTCAATCGCGAAGTGTAGAATGGTCGACGTC	primers for variant specific qPCR
tInactive	32	CAGTCCGGTAAGATAAGCCTGCGATATACCGT	primers for variant specific qPCR
tPV1	32	ATTGACCTGGAGCGCTCGCATGGACACTATTA	primers for variant specific qPCR
tPV2	32	TCAGAGTACGCAGTCAGTCTAATCAGCGACAG	primers for variant specific qPCR
tPV3	32	ACCAGCATTCGAGATGAGGAATCATTCGGTCC	primers for variant specific qPCR
tPV4	32	TTATCCTTACCTCGATCTGGCAACGCAGTCAG	primers for variant specific qPCR
tSDV1	32	ATTAGGAGTAGTGCATCCTGAGCAGCCTGATC	primers for variant specific qPCR
tSDV2	32	GACTCTAAATTACGGCTGATCGACCGAGAGCT	primers for variant specific qPCR
tSDV3	32	CTCATGTCGCCTGATAATATGTGGTGCAGCCA	primers for variant specific qPCR
tSDV4	32	GGTTGGAACAACCTTCTGAGAGTCGCAACTGT	primers for variant specific qPCR
qPCR_rev	30	CAACCGAGCGTTCTGAACAAATCCAGATGG	primers for variant specific qPCR
RTmcr_F	22	CCACAGCTTGCCAAGATCGATG	RT-qpcr expression primers
RTmcr_R	22	ATACTCATCCGCGCCCAGATAG	RT-qpcr expression primers
RTtrf_aF	22	GTGCGAGCTGAAATAGTCGAAC	RT-qpcr expression primers
RTtrfaF_R	22	GAGGAAATCGTCGTGCTGTTTG	RT-qpcr expression primers

Supplementary table 4: List of primers created and used in this study

Supplmentary Table 5 – Statistical tables

Type II ANOVA (Fitness ~ Variant * Colistin Concentration)

	Df	Sum Sq	Mean Sq	F value	Pr(>F)	
Variant	7	1.9362	0.2766	15.332	1.84e-14	***
Colistin Concentration	3	1.0823	0.3608	19.997	1.08e-10	***
Variant: Colistin Concentration	21	0.1676	0.0080	0.443	0.983	
Residuals	128	2.3092	0.0180			

Significance codes: 0 '***' 0.001 '**' 0.01 '*' 0.05 '.' 0.1 ' ' 1

Linear Models

lm(formula = Fitness ~ Expression)

Residuals:

Min	1Q	Median	3Q	Max
-0.093462	-0.065028	0.008597	0.052829	0.100424

Coefficients:

	Estimate	Std. Error	t value	Pr(>|t|)	
(Intercept)	1.41571	0.07779	18.199	3.74e-07	***
Expression	-0.33324	0.08675	-3.841	0.00636	**

Significance codes: 0 '***' 0.001 '**' 0.01 '*' 0.05 '.' 0.1 ' ' 1
Residual standard error: 0.07878 on 7 degrees of freedom
Multiple R-squared: 0.6782, Adjusted R-squared: 0.6323
F-statistic: 14.76 on 1 and 7 DF, p-value: 0.006364

lm(formula = Fitness ~ IC_50)

Residuals:

Min	1Q	Median	3Q	Max
-0.15305	-0.03515	0.00170	0.02978	0.11221

Coefficients:

	Estimate	Std. Error	t value	Pr(>|t|)	
(Intercept)	0.73848	0.11335	6.515	0.000329	***
IC_50	0.27384	0.07606	3.600	0.008738	**

Significance codes: 0 '***' 0.001 '**' 0.01 '*' 0.05 '.' 0.1 ' ' 1
Residual standard error: 0.08224 on 7 degrees of freedom
Multiple R-squared: 0.6493, Adjusted R-squared: 0.5992
F-statistic: 12.96 on 1 and 7 DF, p-value: 0.008738

lm(formula = Fitness ~ Mean Surface Charge)

Residuals:

Min	1Q	Median	3Q	Max
-0.159388	-0.059606	0.000191	0.034426	0.238717

Coefficients:

	Estimate	Std. Error	t value	Pr(>|t|)
(Intercept)	0.8253	0.3527	2.340	0.0519
Mean Surface Charge	0.3949	0.4471	0.883	0.4064

Significance codes: 0 ‘***’ 0.001 ‘**’ 0.01 ‘*’ 0.05 ‘.’ 0.1 ‘ ’ 1
Residual standard error: 0.1317 on 7 degrees of freedom
Multiple R-squared: 0.1003,   Adjusted R-squared: -0.02827
F-statistic: 0.78 on 1 and 7 DF, p-value: 0.4064

__Figures__

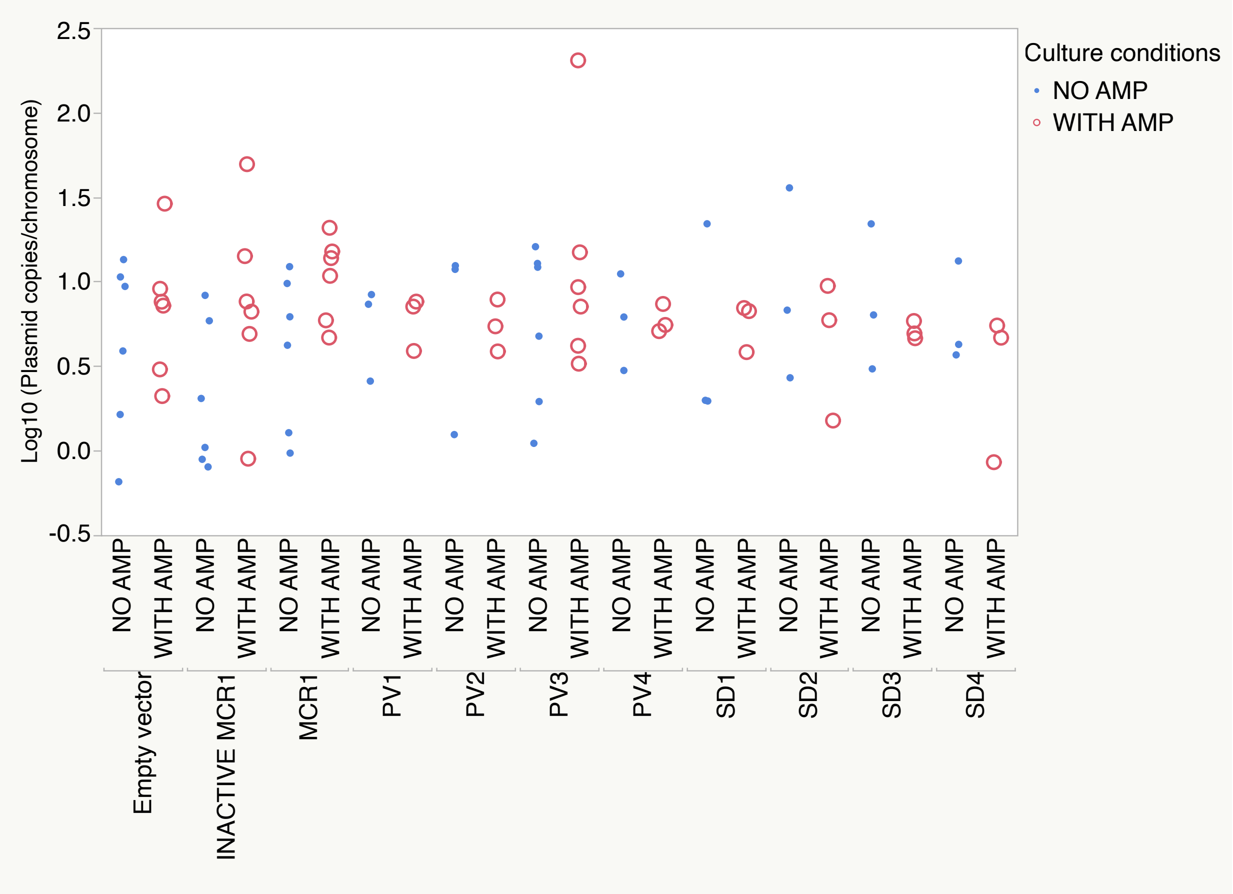

Figure S1. Plasmid stability. Independent colonies of strains used in this study were inoculated into MH+ampicillin (n=3 or n=6 per strain). After overnight incubation, pre-cultures were diluted to an initial titre of approximately 10^5 CFU/mL in MH and MH+ampicillin. Plasmid copy number was determined by qPCR using primers for the pSEVA plasmid (trfA; primers RTtrf_aF, RTtrfaF_R) or a chromosomal control (16S; primers ECfwd, ECrev). qPCR methods were as described in the main text and primer sequences are given in supplementary table 4. No template controls gave high CT values (>30) and all CT values for samples fell within the range covered by standard curves for both primer pairs. Both primer sets gave an amplification efficiency of 100%, allowing us to calculate plasmid copy number as $2^{(\text{CT Chromosomal control-CT Plasmid})}$x7 (16S rRNA copies/chromosome). Plasmid copy number estimates were normalized with a $\log_{10}$ transformation, and we used a 2-way ANOVA to test the impact of strain and culture condition on copy number. Plasmid copy number did not vary between strains ($F_{10,68}$=.41, P=.94), culture conditions($F_{1,68}$=.63, P=.43), or due to the interaction between strain and culture condition ($F_{10,68}$=.88, P=.56). Our overall estimate of pSEVA copy number is 5.58 plasmids/chromosome (95% c.i: 6.76-4.46).

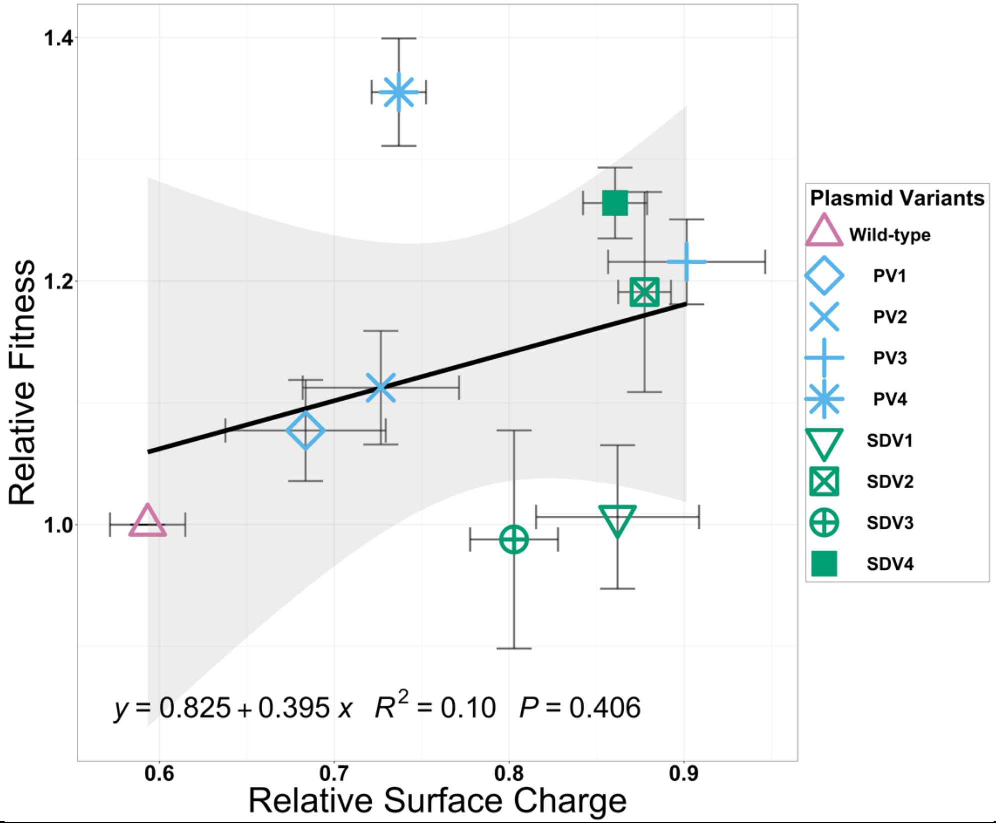

Supplementary Figure S2: Scatterplot showing relationships between relative fitness (y-axis) and relative surface charge (x-axis) of regulatory variants and controls. Points assigned to variants/controls are shown in the legend (error bars = SE). Blank lines show linear regression model fitted to the data with confidence intervals in grey shading. Regression line equations, model r-squared and p-values indicated in black text.

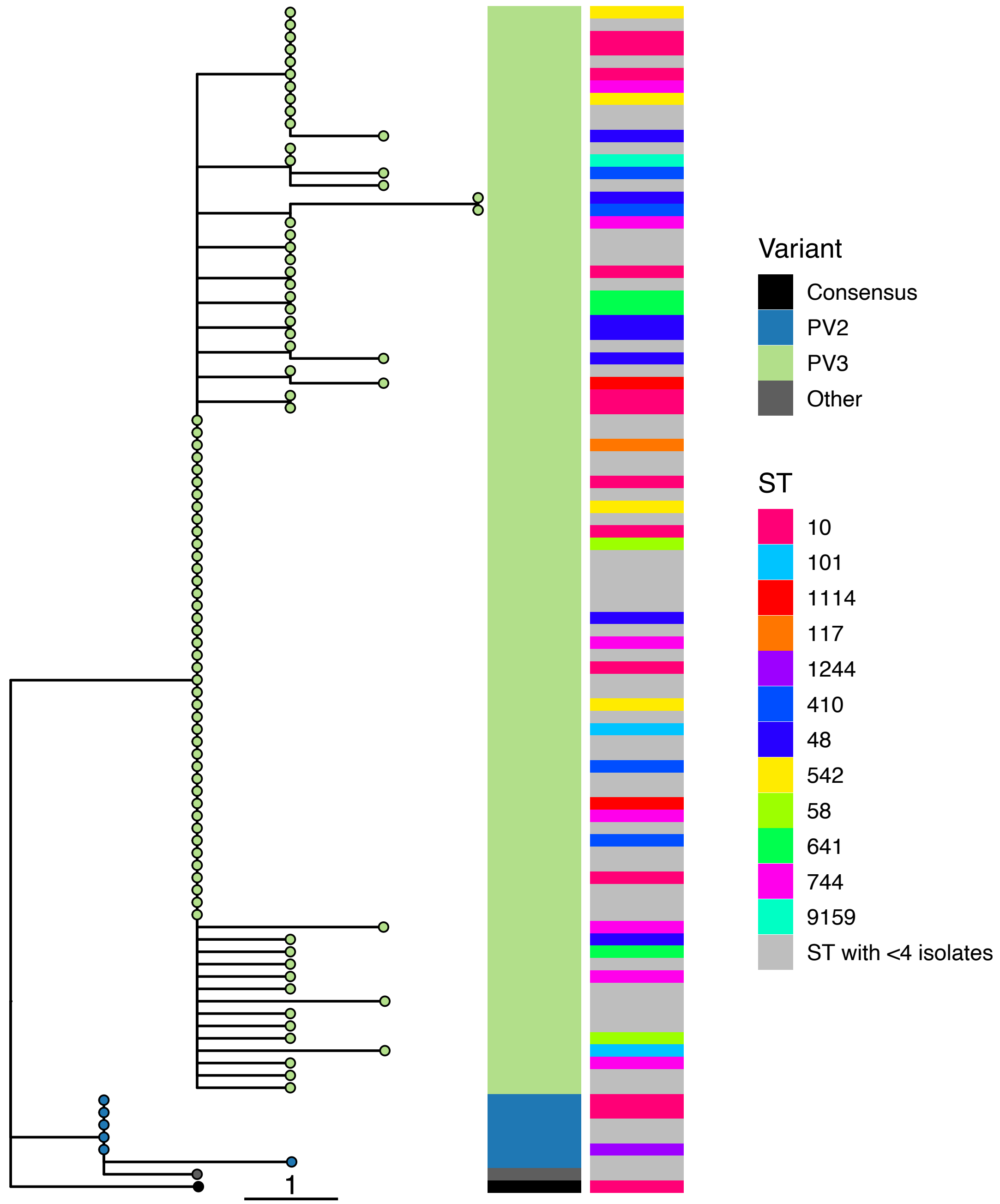

Supplementary Figure S3. A reference-based phylogeny of IncX4 plasmids as in Figure 5C but restricting to only those sampled in 2016. This proves that regulatory variant clades were already present before the colistin ban. Tip colour and first column show regulatory variant, second column shows ST (STs with <4 IncX4 plasmids in the whole dataset are coloured grey; colours as in Figure 5C).

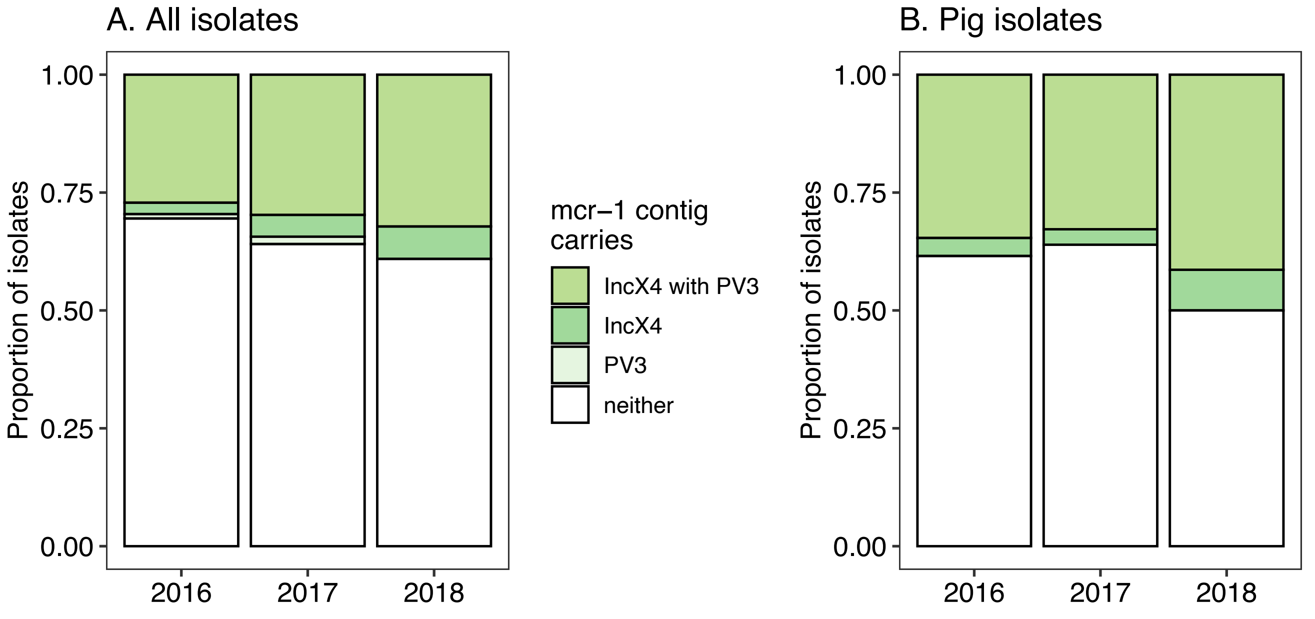

Supplementary Figure S4: Increase in proportion of isolates carrying an IncX4 plasmid with PV3 from a genomic dataset of *mcr-1*+ isolates collected pre- and post-colistin ban. **A**) All isolates where the de novo assembly had an *mcr-1*-carrying contig (n=674). **B**) The subset of those isolates which were collected from pigs (n=197). Isolates are grouped by whether the *mcr-1*-carrying contig had a single IncX4 replicon and/or the PV3 regulatory mutation. Contigs with IncX4 and other replicons were not included within "IncX4", because we are interested in the spread of plasmids similar to the reference plasmid which carries only IncX4.

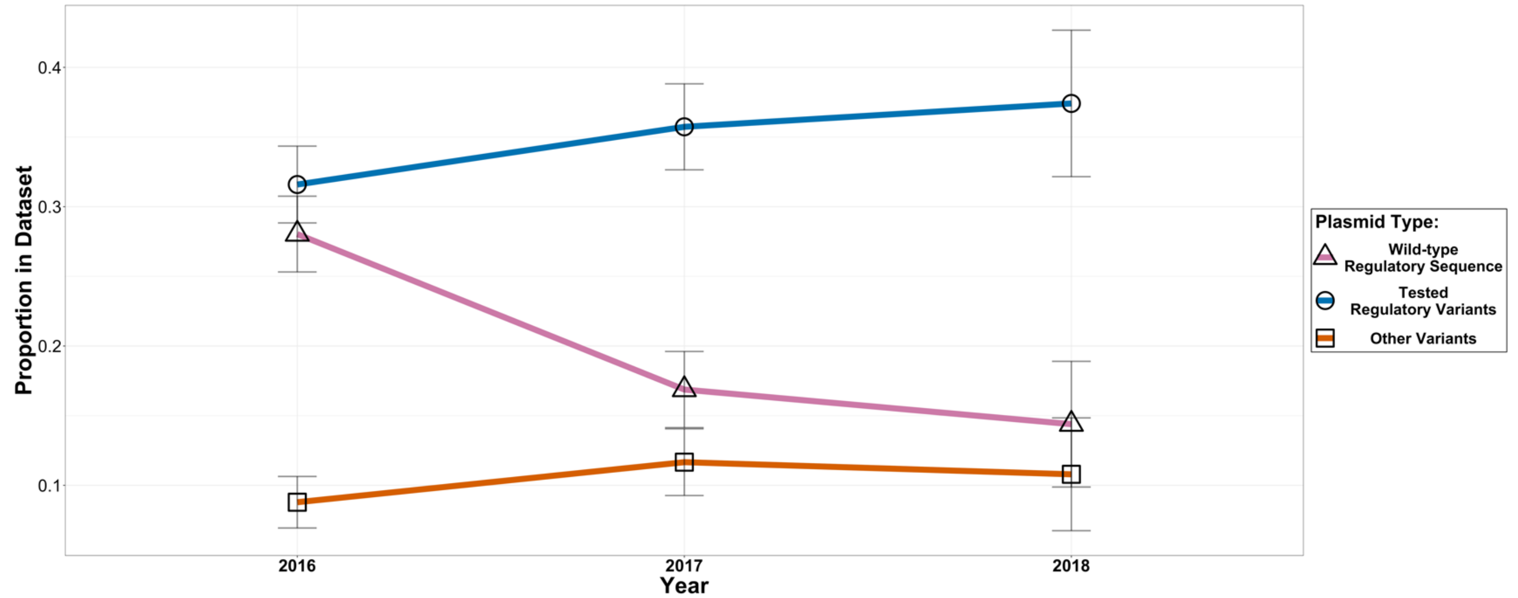

Supplementary Figure S5: Proportion of WT (pink) tested variants (blue) and other (green) regulatory variant sequences in Shen dataset as separated by year. Error bars show the standard error of proportions.
